# Briquetting of subbituminous coal and torrefied biomass using bentonite as inorganic binder

**DOI:** 10.1038/s41598-022-12685-5

**Published:** 2022-05-24

**Authors:** A. A. Adeleke, J. K. Odusote, P. P. Ikubanni, A. S. Olabisi, P. Nzerem

**Affiliations:** 1 Department of Mechanical Engineering, Nile University of Nigeria, Abuja, Nigeria; 2 Department of Materials and Metallurgical Engineering, University of Ilorin, Ilorin, Nigeria; 3 Department of Mechanical Engineering, Landmark University, Omu-Aran, Nigeria; 4 Department of Mechanical Engineering, William Tubman University, Harper, Liberia; 5 Department of Petroleum and Gas Engineering, Nile University of Nigeria, Abuja, Nigeria

**Keywords:** Energy science and technology, Engineering, Materials science

## Abstract

The use of inorganic binder for briquetting of subbituminous coal and torrefied biomass for energy generation is scarce. The present study focuses on the physicomechanical durability and energy content of briquettes produced from subbituminous coal (SubC) and torrefied biomass (TM) using bentonite as binder. Briquettes were produced using 95% SubC and 5% TM. Bentonite was varied at 2–10% of the total SubC and TM weight. The briquettes were produced with a constant pressure (28 MPa) in a hydraulic press. The briquettes were primarily cured at room temperature and then at 300 $^\circ{\rm C}$ in a tubular furnace under an inert condition for 60 min. The density and water resistance (WRI) of the briquettes were evaluated. Drop to fracture (DF), impact resistance index (IRI), cold crushing strength (CCS) and tumbling strength index (TSI_+3 mm_) of the briquette were obtained. The reactivity index (RI), proximate, ultimate and calorific values analyses were assessed based on different ASTM standards. Microstructural studies and elemental mapping were carried out using scanning electron microscope equipped with EDS and electron probe microanalyzer. The density increased with increment in bentonite content. The WRI decreased with increase in bentonite while the least (95.21%) was obtained at 10% binder content. The DF and IRI ranges from 100 to 150 and 2000–3000, respectively. The CCS were in the range of 19.71 to 40.23 MPa. The RI varies from 34 to 50%. Fixed carbon, carbon and calorific values were impaired as the bentonite content in the briquette increases. Oxygen and silica bridges with mechanical interlocking were observed on the micrographs of the briquettes. The briquettes produced with 2% bentonite content have better physicomechanical durability with equivalent energy content. It is recommended as feedstock for thermal and metallurgical applications.

## Introduction

Waste generation is an integral part of man. Some of these wastes are good raw materials for various industrial and domestic applications. Wastes from coal mining, handling and transportation are always in million tonnes^1^. Coal fines (< 3 mm) are often referred to as wastes and are inevitably produced when lump coals are processed or handled^2,3^. Wastes from the wood processing industries have also been reported to be in million tonnes especially in developing countries^4,5^. These wastes have been found useful in various area of applications which includes energy generation^6,7^, reinforcement in metal matrix composites^8–10^, microelectromechanical systems^3^ among others. Predominantly, developing nations have peculiar issues with low energy mix. Thus, researchers from various fields continue to harness these wastes (coal and biomass) as possible additional energy sources to the existing ones. Adeleke^11^ improved the energy content of biomass wastes through mild pyrolysis and added it to lean grade coal wastes to produce composite briquettes. The fuel briquettes produced were recommended for industrial and domestic usage. Adeleke et al.^12^ produced briquettes from upgrade biomass and coal fines as solid fuel. It was reported that the briquettes were mechanically stable with good combustion characteristics. Trubetskaya et al.^13^ characterized woodstove briquettes from torrefied biomass and coal. Inorganic matter influenced the reactivity of the briquettes less than the organic composition of the raw materials. The porosity of the briquettes was lowered with increase in the inorganic matter. Physicomechanical integrities of the briquettes were not reported. Guo et al.^14^ optimized composite binders for lignite briquettes. The binders used were polyvinyl alcohol and sodium humate. Sodium humate (2wt.%) and polyvinyl alcohol (0.5wt.%) were obtained as the optimal composite binder for better mechanical strength. The lignite briquettes were recommended for industrial applications. In an attempt to produce strong briquettes from coal wastes, molasses and coal tar pitch were blended as binder by Zhong et al.^15^. The best briquette produced was reported to have a compressive strength of 13.06 MPa with a drop to fracture of 56.6 time/2 m. The briquettes were primarily produced for COREX iron making processes. Adeleke et al.^2^ produced and characterized composite briquettes from coal and wood fines using pitch binder. The wood fines were initially torrefied for an improved calorific value and enactment of its bonding properties. The briquettes were produced from 3 to 20% torrefied biomass and 80–97% coal fines. The optimum cold crushing strength of 4 MPa, drop to fracture of 54 time/2 m and an impact resistance index of 1350 were recorded for the composite briquettes. The briquettes were recommended for industrial application. Adeleke et al.^4^ further produced briquettes from torrefied biomass and coal where molasses and blended pitch were used as binders. The tumbling strength index (TSI_+3 mm_) and reactivity index (RI) of the samples were evaluated for possible use as feedstock in metallurgical applications. The TSI_+3 mm_ was obtained for the cured samples and samples exposed to 1200 $^\circ{\rm C}$. The TSI_+3 mm_ of the cured briquette samples were between 95.5 and 98.3% which drastically declined to 57.4–77.4% for the samples were exposed to 1200 $^\circ{\rm C}$. The RI of the briquettes were between 48 and 56%, and it was an indication of high reactiveness. As a result of the TSI_+3 mm_ and RI, the briquettes were reported to be appropriate as carbonaceous material especially in rotary kiln in the making of direct reduced iron. There is an unending argument about the mechanical stability of various briquettes produced as composite of coal and biomass. This led to a renewed interest in using various type of binders to produce briquettes with better mechanical strength without compromising its energetic value. This could ultimately guide researchers and industrialists to standardized acceptable mechanical and energy properties of solid fuel briquettes. Thus, the present study focuses on improving the mechanical integrity of briquettes produced from subbituminous coal and torrefied biomass using bentonite, which is an inorganic binder. Bentonite is an aluminum phyllosilicate which is obtained frequently from volcanic ash alteration. This binder is available in million tons in Nigeria^16^. Bentonite is a good binder with tendency of improving the strength of briquettes with no addition of pollutant to the composite materials^17^. The present study is proposed based on limited research work on the use of bentonite as binder for briquetting of subbituminous coal and torrefied biomass. Briquettes are produced from subbituminous coal (95%) and torrefied biomass (5%) while varying bentonite based on the total weight of the briquettes from 2 to 10%. Physicomechanical and energy content analyses were carried out on the briquettes. The use of bentonite as inorganic binder is expected to improve the physicomechanical properties of the hybrid briquettes. This will serve as good comparison for briquettes produced from other organic and inorganic binders.

## Methodology

### Materials

**Figure 1:**
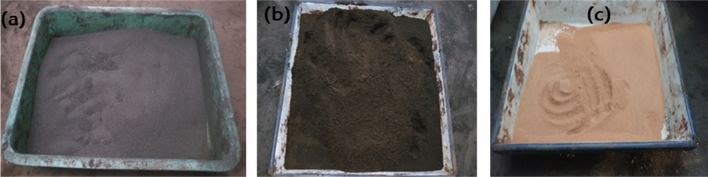
Raw materials (**a**) subbituminous coal, (**b**) torrefied biomass, (**c**) bentonite.

The materials used for the production of briquette in this study were subbituminous coal (SubC) fines, melina woody biomass (MWB) and bentonite. SubC was obtained from Okaba mine, Nigeria while MWB was obtained from Benin City, Nigeria. Bentonite was used as binder and it was obtained from Jamshedpur, India. These raw materials are shown in Fig. 1.

### Material preparations

Subbituminous coal fines were further pulverized, sun-dried and screened to less than 0.70 mm. Further drying was done in an oven at 105 $^\circ{\rm C}$ for 30 min to eliminate unbounded moisture as previously described by Adeleke et al.^1^. The proximate, ultimate and calorific value (HHV) as reported by Adeleke et al.^2^ are shown in Table 1. The details of torrefaction of melina has also been reported by Odusote et al.^7^. The torrefied biomass used was below 0.70 mm. Table 1 showed the proximate, ultimate, and calorific values of the torrefied biomass. The bentonite was sun-dried and screened to particle size below 0.70 mm. This was to achieve uniformity in particle sizes for all the composite materials and binder. The chemical composition (oxides) of the bentonite was obtained using X-ray fluorescence spectrometer (Bruker 58 TIGER model). The compositions are presented in Table 1.

**Table 1:** Properties of raw materials for production of briquette.

	Proximate analysis (%)				Ultimate analysis (%)					(MJ/kg)
	MC	VM	AC	FC	C	H	N	S	O	HHV
SubC	1.37	13.71	18.00	64.92	71.47	2.88	0.90	0.71	24.04	24.20
TM	2.63	54.07	2.17	41.08	66.08	5.18	0.30	0.20	26.30	23.45

	Chemical compositions (%)									
	SiO_2_	Al_2_O_3_	Fe_2_O_3_	CaO	Na_2_O	MgO	K_2_O	TiO_2_	P_2_O_5_	Others
Bentonite	51.02	10.28	3.42	1.21	1.14	3.04	1.56	0.64	0.89	–

*MC* moisture content, *VM *volatile matter, *AC* ash content, *FC* fixed carbon, *C* carbon, *H *hydrogen, *N* nitrogen, *S* sulphur, *O* oxygen, *HHV* calorific value, *TM* torrefied biomass and *SubC* subbituminous coal.

### Briquetting

Subbituminous coal fines (95% of 25 g), torrefied biomass (5% of 25 g) and bentonite (2–10% of the overall briquette weight) were mechanical mixed. Water was added as 10% overall weight of coal and torrefied biomass, and the whole materials were blended together using a mechanical stirrer at 50 rpm for five (5 min) in order to obtain homogeneousness. The blend was then dispensed a cylindrical die of 25 mm internal diameter. A hydraulic press at a constant pressure of 28 MPa was used to compress the blend to briquettes. The load was gradually removed from the die and then the sample was ejected from the mold. The green briquettes were allowed to dry at room temperature for 36 h. Further curing of the samples was done by introducing nitrogen (50 ml/min) into a tubular furnace to form an inert environment at a temperature of 300 $^\circ{\rm C}$ for a 60 min residence time. The samples were removed and placed in a desiccator to be cooled at room temperature. The samples were preserved in a zip-lock bag prior to physicomechanical integrity and energy content assessment.

### Physical integrity

The physical integrity is adjudged with the physical properties such as density and water resistance index (WRI). The densities of the briquettes were obtained using Eq. (1), where m is mass and v is volume. The water resistance was obtained using modified Richard's method^18^. Briquette with weight (${\mathrm{W}}_{1}$) was immersed in a cylindrical glass which contains distill water of volume 200 ml at $30\pm 2\mathrm{^\circ{\rm C} }$ for 30 min. The briquette sample was then removed, cleaned to reduce water on its surface. The sample was later reweighed as ${\mathrm{W}}_{2}$. The relative change in weight of the briquette was determined and percentage water absorption was evalulated using Eq. (2) while WRI (%) was obtained using Eq. (3).

1 $$\mathrm{Density}=\frac{\mathrm{m}}{\mathrm{v}}$$

2 $$\mathrm{Water\, absorption }\left(\mathrm{\%}\right)=\frac{{\mathrm{W}}_{2}-{\mathrm{W}}_{1}}{{\mathrm{W}}_{1}}$$

3 $$\mathrm{WRI }\left(\mathrm{\%}\right)=100-\mathrm{\%water\, gained}$$

### Mechanical integrity

The mechanical integrity of briquette is a measure of the mechanical properties of the briquettes. These include cold crushing strength (CCS), drop to fracture (DF), impact resistance index (IRI) and tumbling strength index (TS1_+3 mm_). A universal mechanical testing machine (10 Kw Hounsfield apparatus) was used to obtain the CCS. The compression mode of the machine was used as stipulated for coke and briquettes^19^. The maximum crushing load (${\mathrm{M}}_{\mathrm{f}}$) the briquette can bear prior to cracking was noted and was done in triplicates for each briquette. The average ${\mathrm{M}}_{\mathrm{f}}$ was then utilized to determine the CCS based on Eq. (4). In Eq. (4), D is the bottom circular diameter for the briquette. DF was carried out by dropping briquette sample from a height of 2 m until it breaks. The average times/2 m taken for it to break was noted. The average of three replicates were utilized to evaluate the drop resistance. IRI was obtained from the DF test using Eq. (5).

4 $$\mathrm{CCS}=\frac{4{\mathrm{M}}_{\mathrm{f}}}{\uppi {\mathrm{D}}^{2}}$$

5 $$\mathrm{IRI}=\frac{100\times \mathrm{Average\, number\, of\, drops}/2\mathrm{m}}{\mathrm{Average\, no\, of\, pieces}}$$

The tumbling strength index (TS1_+3 mm_) for the briquettes was obtained using the method reported in the study of Adeleke et al.^4^. Some samples were exposed to 1200 $^\circ{\rm C}$ in a furnace and held for 2 h. The cured and those exposed to 1200 $^\circ{\rm C}$ were adopted for the tumbling test. Three briquette samples of identified weight (${\mathrm{W}}_{\mathrm{o}}$) were placed in a steel tube (40 mm inner diameter, 200 mm length) and were allowed to rotate at a speed of 30 rpm for 20 min. After tumbling, the samples were removed and then screened on a 3.15 mm sieve size. The + 3 mm particles of the sample were weighed. The obtained values were used to evaluate the TS1_+3 mm_ in accordance with Eq. (6).

6 $${TSI}_{+3mm}=\frac{{W}_{+3mm}}{{W}_{o}}\times 100$$

where ${W}_{+3mm}$ and ${W}_{o}$ are the weight of + 3 mm particle sizes and initial samples, respectively.

### Proximate, ultimate, calorific values and reactivity index analyses

The reactivity of the briquette samples was carried out in accordance with ASTM D5341M-14 standard^20^. The details of this method have been reported in our previous study^2^. The RI was obtained in duplicates for each sample. Proximate analyses project moisture content (MC), ash, volatile matter (VM) and fixed carbon (FC) contents of the pulverized samples and it was carried out following the IS: 1350-1 standard^21^. The ultimate analysis (Carbon, Hydrogen, Nitrogen, Sulphur and Oxygen) for the pulverized briquette was carried out based on ASTM D5373-16 standard^22^ while the calorific value was obtain in accordance with ASTM D5865-04 standard^23^ using Oxygen Bomb Calorimeter (Model A1290DDEE).

### Microstructural studies and elemental mapping

The microstructures of the briquettes were observed under a scanning electron microscope (Nova Nano SEM 430) equipped with EDS. The briquette with 2% bentonite was exposed to elemental mapping under the electron probe microanalyzer equipped with EDX (JEOL 8230 Model). This was because it gave the best energy value. Thus, the need to understand the spread and coverage of each element within its formation.

## Results and discussion

### Density and water resistance of the briquettes

**Figure 2:**
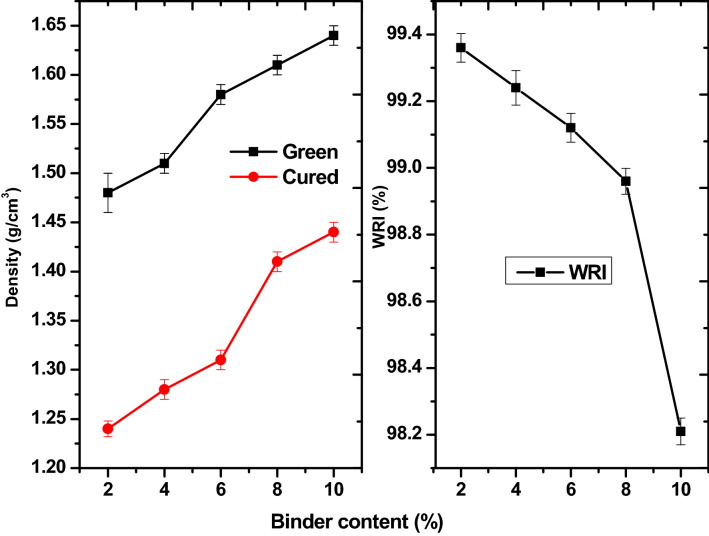
Physical properties of the fuel briquettes (**a**) density, (**b**) WRI.

Density is a vital physical property of fuel briquettes. Higher density implies higher energy/volume ratio. The densities of the green and cured briquettes are shown in Fig. 2a. The densities of green samples were in the range of 1.48 to 1.64 g/cm^3^. The densities of the cured briquettes were between 1.24 and 1.44 g/cm^3^. The density of the briquette increased with increase in bentonite content. This implied that bentonite is denser and thus, an increase in density. The curing process led to a reduction in density. This is expected as unbounded moisture loss, light volatiles evolution and reactive drying takes placed at 300 $^\circ{\rm C}$^24^. The density of the briquettes produced in this study is a little higher than our previous studies^1,2^. This is because of the addition of bentonite, which is denser than the binders (molasses and pitch) used in those studies. Materials with finer particles possess large surface area for bonding. This may also be responsible for the higher density since bentonite is finer by nature than pitch binder. More of bentonite in briquettes could further make briquette denser. While there are no standard acceptable values for briquettes, higher density is good for transportation as it lowers cost and elongate burning time^25^. However, the combustion properties of briquettes with very high density are negatively impacted. Thus, a need for balance. Richard^18^ is a widely acceptable reference for the properties of briquettes. The density recommended for acceptable briquettes of industrial and domestic use ranges from 1.25 to 1.30 g/cm^3^. Briquettes with 2 and 4% bentonite met this requirement. The briquettes produced in this study are fit for transportation, handling and storage. The water resistance index (WRI), shown in Fig. 2b, indicates the degree at which briquettes can withstand degeneration in humid environment. The WRI for the briquettes range from 98.21 to 99.36%. It could be observed that increase in bentonite led to a continuous reduction in WRI. This indicates that bentonite is hydrophilic in nature. More bentonite content implied that more water would be absorbed and retained in the briquette. The WRI of the present briquettes compare well with the works of Mollah et al.^26^, Zhong et al.^15^ and Adeleke et al.^4^. Richard^18^ benchmarked WRI for acceptable briquette as 95%. By implication, all the briquette samples produced surpasses the benchmark. However, higher bentonite contents could lead to higher moisture attraction. This can lead to partial or total disintegration of the briquettes when exposed to humid condition or in contact with water. Though the WRI for the briquettes compares well briquettes recommended for various energy applications, it should be stored in a place with little or no exposure to moisture.

### Drop to fracture and impact resistance for the briquettes

**Figure 3:**
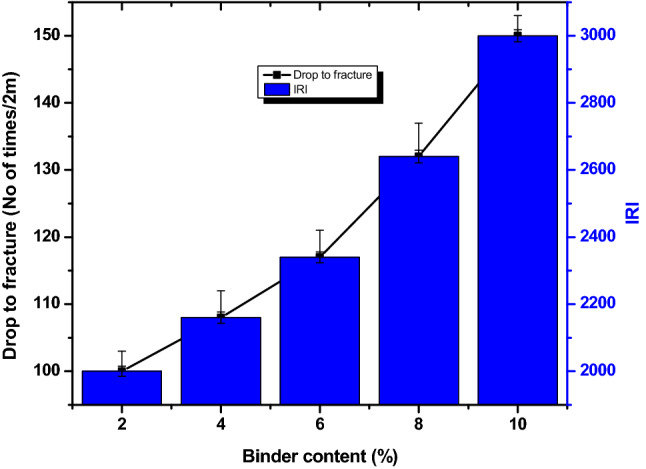
Drop to fracture and IRI for the fuel briquettes.

The response of briquette to gravitational deterioration is an indication of its mechanical durability^27^. Drop to fracture (DF) and impact resistance index (IRI) are useful tools in evaluating the durability. The DF and IRI for briquettes in this study are shown in Fig. 3. The DF varies from 100 to 150 times/2 m and the highest was obtained at 10% bentonite content within the briquette. Bentonite contains high SiO_2_, which implied that the low temperature bonding strength of Si–O–Si bonds might have strengthened the briquettes against gravitational impact. The IRI values for the briquettes vary from 2000 to 3000. This range of value is extremely high compared to IRI of 50 that was recommended for briquettes of industrial applications^18^. The IRI of the briquette produce using bentonite binder is higher than what was obtained for briquettes of coal and biomass using pitch and molasses binders (150–1175) as reported by Adeleke et al.^2^. The curing process for the briquettes interestingly enacted the bonding strength of Si–O–Si bonds within the bentonite at 300 $^\circ{\rm C}$ to enhance the DF and IRI. All the briquettes are very good feedstock that can be transported, handled and stored based on the DF and IRI without degeneration.

### Cold crushing strength of the briquettes

**Figure 4:**
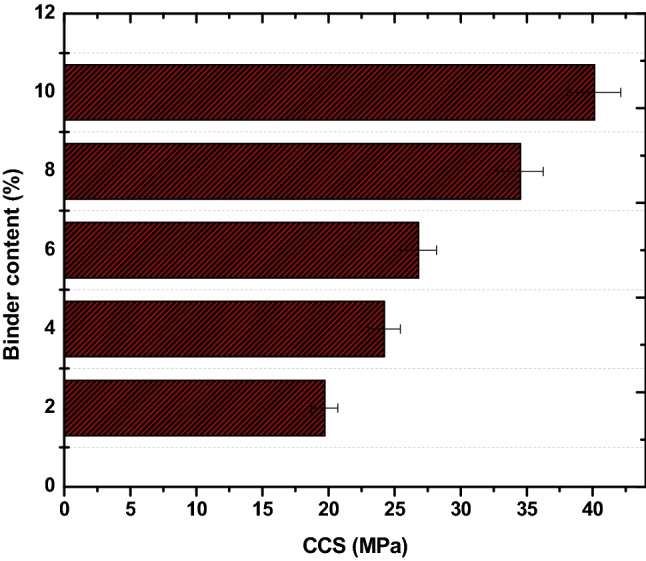
CCS of the fuel briquettes.

Figure 4 shows the cold crushing strength (CCS) for the briquettes. The CCS depicts the ease of breakage or wear during transportation, handling and storage of the briquettes. CCS is also a litmus check of the mechanical durability of the briquettes. The CCS of the briquettes produced in this study were in the range of 19.72 to 40.12 MPa. The CCS increased with bentonite increment in the briquette. As earlier stated, the present study explored the Si–O–Si bonds reported as strong bond for geopolymer making at low temperature to enhance the CCS of the briquettes^4^. The more the Si–O–Si bonds in the briquettes, the better the CCS. Comparatively, the briquette outperformed all our previous studies on coal and torrefied biomass briquettes in terms of CCS^2,28^. The briquette strength surpassed the minimum 1.0 MPa recommended by Borowski and Hycnar^29^ for briquettes of industrial applications. The DF, IRI and CCS of the briquettes were positively influenced by increase in bentonite. The physicomechanical properties of the briquettes shows they are essentially durable and fit for transportation, handling and storage conditions. Thus, bentonite is a viable inorganic binder for briquetting subbituminous coal and torrefied biomass to durable fuel.

### Tumbling strength index for the briquettes

**Figure 5:**
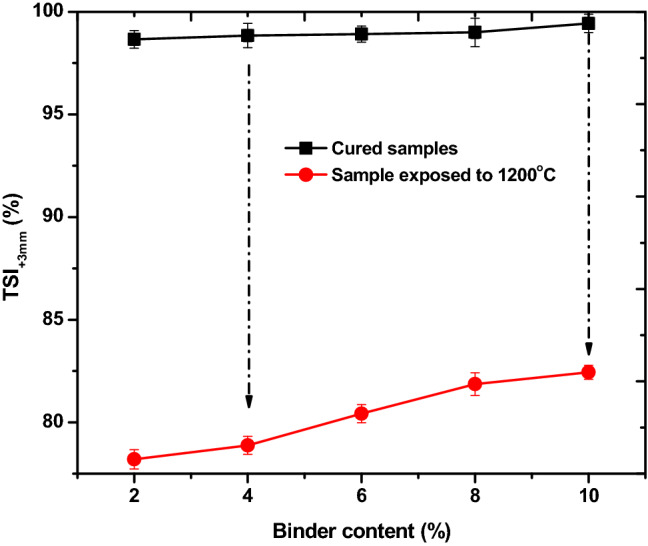
Tumbling strength index of the produced fuel briquettes.

Figure 5 shows the tumbling strength index (TSI_+3 mm_) for all the briquettes. The tumbling strength is referred to as attrition strength and it is measured through the TSI_+3 mm_ values. For all the briquettes, the tumbling strength indices surpassed the 95% recommended by Richard^18^ and Thoms^30^ for durable briquettes. The responses of the briquettes in this study to attrition forces is slightly similar to the briquettes produced using pitch and molasses as binder. There is an improvement in TSI_+3 mm_ for the briquettes in the present study. This may be due to bonding strength of the content of bentonite (SiO_2_, MgO and CaO). The TSI_+3 mm_ of the cured briquettes were extremely attractive (> 95%) and this implied less generation of small particles (fines) under tumbling forces or attrition during handling, transporting and utilizing the briquette. The TSI_+3 mm_ of the samples exposed to 1200 $^\circ{\rm C}$ was in the range of 78.20 to 84.44%. The TSI_+3 mm_ is a mimic of coke strength after reduction (CSR) for coke. A CSR of 65% is an indication of low reactivity, which is good for coke^31,32^. Compared to briquette samples that were only cured before tumbling test, further devolatilization and degradation of subbituminous coal and torrefied biomass is expected to reduce the TSI_+3 mm_ of those exposed to 1200 $^\circ{\rm C}$. Thus, the rationale behind the reduced TSI_+3 mm_. The tumbling strength index at 1200 $^\circ{\rm C}$ is required for briquettes produced with the intention of dual purposes (energy feedstock in thermal plants and metallurgical reductant). Thus, the tumbling strength of the present briquettes indicates that they will have resistance to tumbling degradation under high temperature regime within a rotary kiln. The briquettes are suitable for metallurgical process in kilns.

### Reactivity index of the briquettes

**Figure 6:**
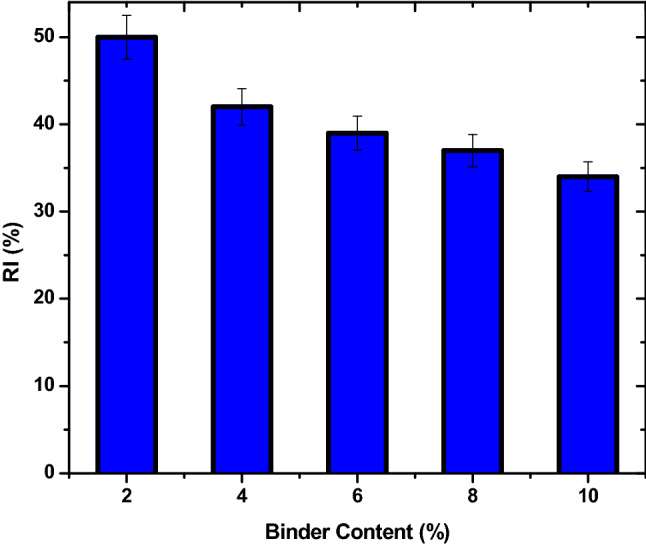
Influence of binder variation on reactivity index (RI) of the hybrid fuel briquettes.

The reactivity indices (RI) of the briquettes are presented in Fig. 6. The RI of the briquettes was in the range of 34 to 50%. The least reactive was the sample produced with 10%. The higher the bentonite content, the lower the RI. RI in its essence indicates the rate of reactive performance and mass loss tendency for the briquettes especially in oxidizing environments. It is expected that in use, briquettes will experience losses in weight and contents because of devolatilization and degradation. However, it must not be excessive^5^. The decrease in RI of the briquettes due to increase in bentonite is an indication of its extremely low reactivity^16^. The RI of the samples were above the 20–30% recommended range for normal coke used in blast furnace as fuel and reductant^33^. The essence of the test is to understand the reactive behaviour of briquettes made with bentonite binder. The test has shown that the briquette will react well with other feedstock in a reduction scenario in a rotary kiln at $\le$ 1200 $^\circ{\rm C}$.

### Proximate and ultimate contents, and calorific values of the briquettes

**Figure 7:**
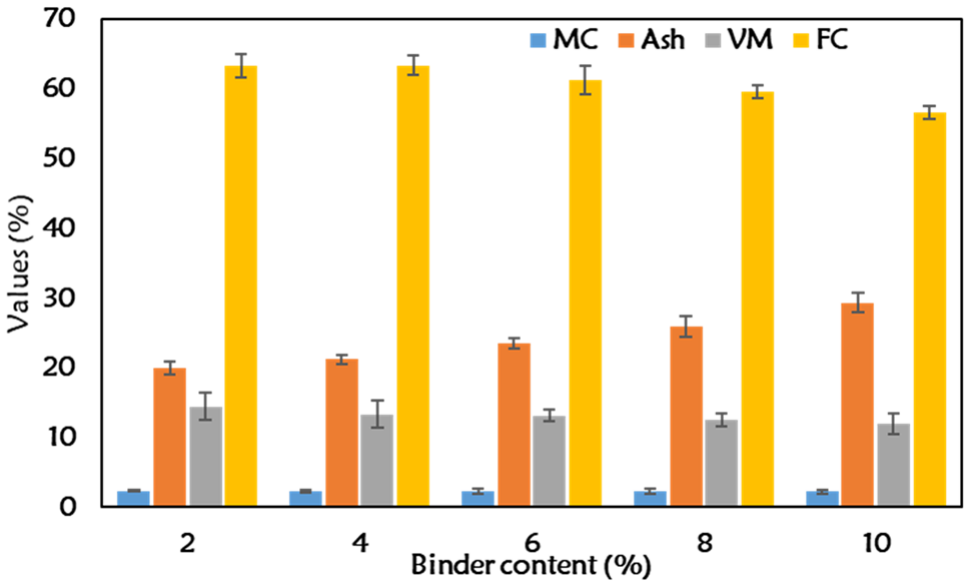
Proximate analyses of samples with varying binder contents.

The inorganic binder used for the production of briquettes produced in this study has been opined to drastically affect its energy content negatively^34^. Thus, the proximate, ultimate and calorific values of the briquette are the major litmus check of its energy content and usefulness. Chou et al.^35^ and Ajimotokan et al.^36^ emphasized that briquettes with good physicomechanical properties and poor energy content makes poor solid fuel. The proximate content is presented in Fig. 7. An increase in bentonite led to reduction in fixed carbon (FC), increase in ash while volatile matter and moisture were constant. The reduction in fixed carbon indicates largely a reduction in calorific values (heating values). This is true for these briquettes as HHV reduced from 24 to 17 MJ/kg for briquettes with 2% to 10% bentonite, respectively. Fixed carbon is a major indicator of how efficient solid fuel is for energy and metallurgical applications^37^. The presence of SiO_2_ and other inorganic oxides in bentonite plays a significant role in the declined energy content. However, with 2% bentonite, the briquettes displayed similar properties reported in our previous study^2^. The FC of the briquettes produced from coal and biomass using organic binders were in the range of 65.13 to 65.25%. Increase in bentonite content damaged the energy content of the briquettes and will affect its combustion behavior in use. The carbon (C), hydrogen (H), nitrogen (N), sulphur (S) and oxygen (O) contents for the briquettes are presented in Table 2. Notably, the carbon reduced with increase in bentonite from 72.74 to 63.41%. This is expected since the FC had a decline. The H, N, S were nearly constant while oxygen also reduced with increase in bentonite content. There is higher tendency of more Si–O–Si bond with oxygen as bentonite increased. Thus, an increased bounded oxygen by chemical reaction. From all indications, increased bentonite within the briquette impaired more energy content. Thus, 2% bentonite that produces enviable mechanical durability in the briquettes is enough for bonding the subbituminous coal and torrefied biomass as solid fuel.

**Table 2:** Ultimate contents and calorific values of the briquettes.

%Binder content	%C	%H	s%N	%S	%O	HHV (MJ/kg)
2	72.74	2.46	0.91	0.71	23.18	24.00
4	71.72	2.44	0.90	0.70	24.24	22.54
6	68.50	2.44	0.89	0.71	27.46	21.12
8	67.66	2.45	0.90	0.71	28.28	19.84
10	63.41	2.42	0.90	0.71	32.56	17.68

### Microstructural study and elemental mapping for the briquettes

**Figure 8:**
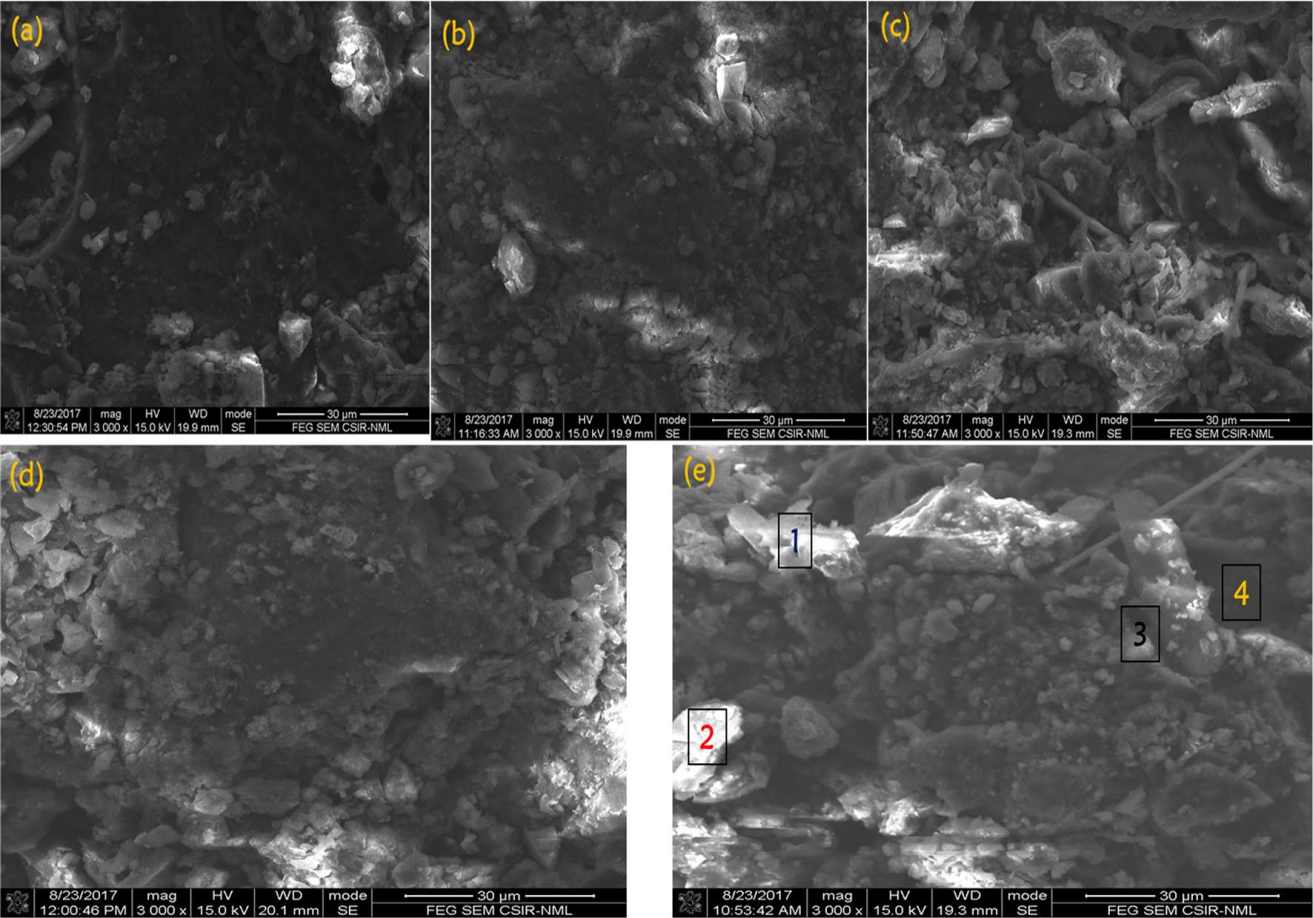
SEM images of the briquettes with varying bentonite contents (**a**) 2%, (**b**) 4%, (**c**) 6%, (**d**) 8%, (**e**) 10%.

**Figure 9:**
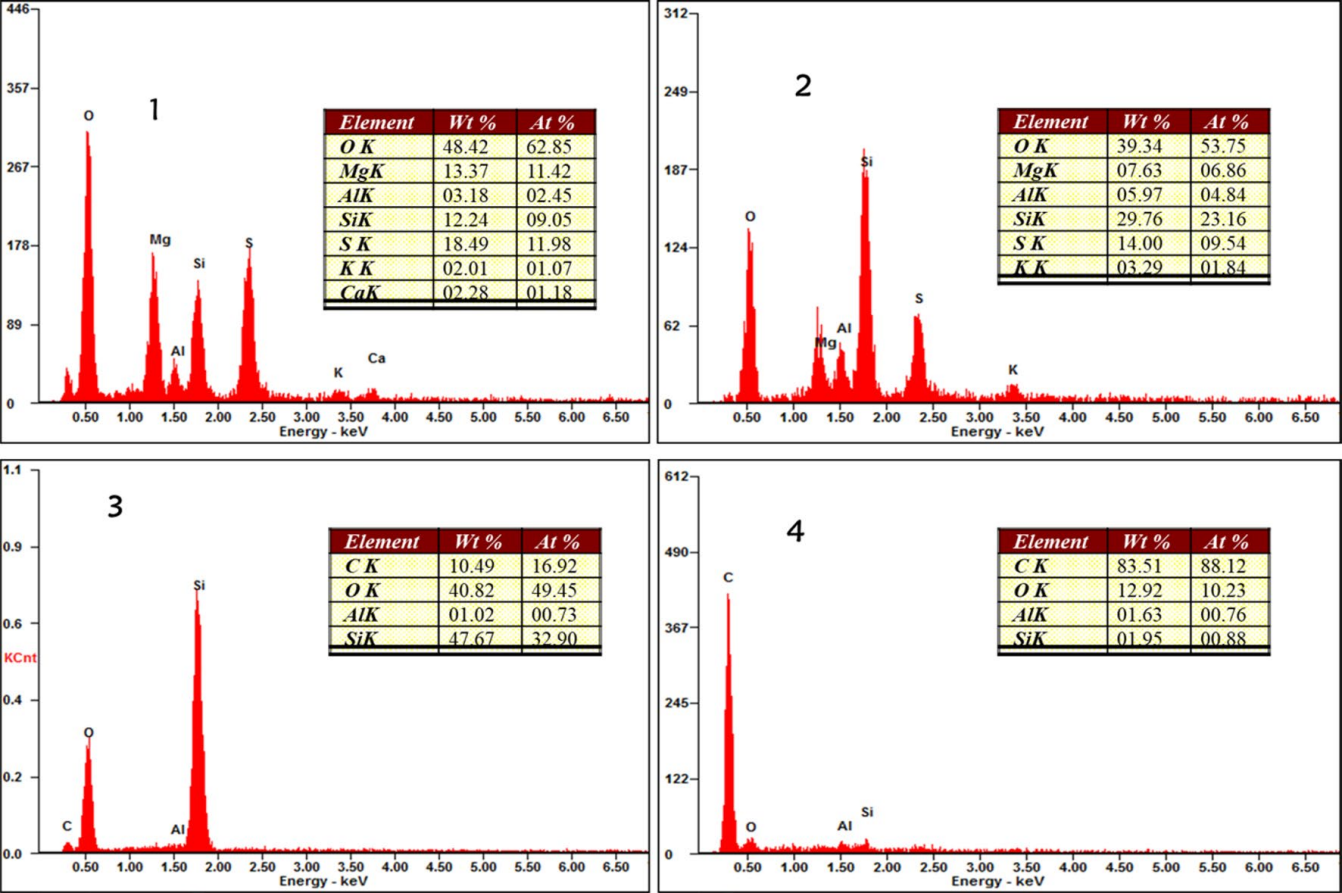
Point elemental analyses of sample with 10% bentonite.

**Figure 10:**
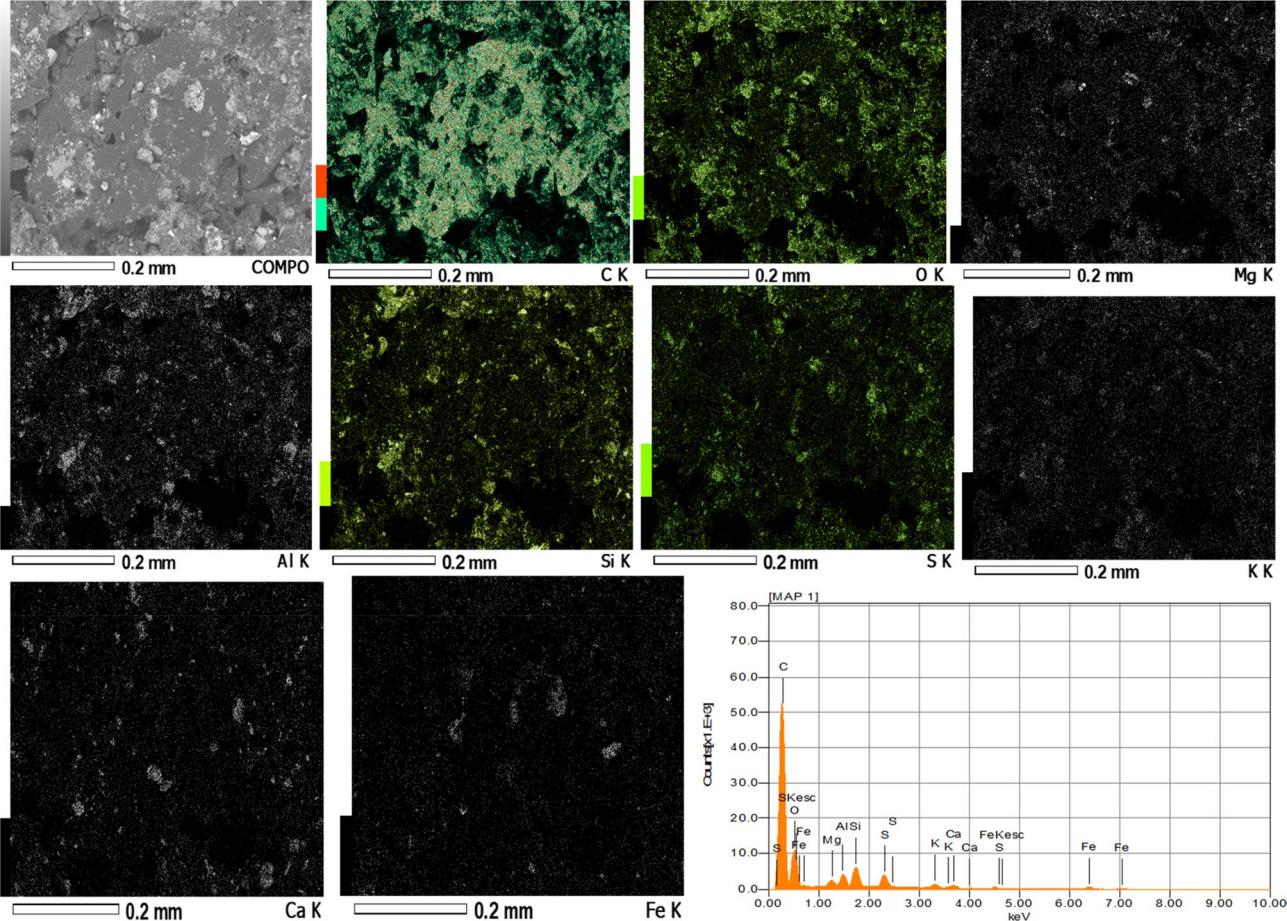
Elemental mapping of the briquette with 2% bentonite.

To understand the mechanism of bonding, briquette samples were observed under microscope and the SEM images are presented in Fig. 8. The images (Fig. 8a–e) display granular and irregular structure with some charging effects. The charging effect was reported by Zhong et al.^15^ for coal briquettes and it increases with increased bentonite within the briquettes in this present study. Figure 8e shows more of this microstructure. This phenomenon has been adduced to be oxygen bridges in previous studies^5^. However, with the use of bentonite as binder, this may be oxygen-silica bridges. The oxygen-silica bridges were pronounced on Fig. 8d,e. Coupled with mechanical interlocking that could be seen in the structural makeup of the briquettes, the oxygen bridges and silica content may be responsible for improved strength with increased bentonite. In a critical evaluation, Fig. 9 projects the elemental analysis of four different points on the SEM image of Fig. 8e. Oxygen and silicon dominated the area where the charging effects were pronounced (1, 2, and 3) while the dark spot (4) contained more carbon content (83.51%). This is an addendum to initial explanation that silica plays a significant role along with oxygen bridges in improving strength of the briquettes. The elemental mapping of the best briquette in terms of energy content (2% bentonite) is presented in Fig. 10. The mapping shows that carbon is the predominant element in the briquette. This is because subbituminous coal and biomass are majorly carbon dominated. Figure 10 also shows that oxygen, magnesium, aluminum, silicon, sulphur, potassium, calcium and iron were picked along carbon. The uniform spread of these elements is important for complete combustion when briquettes are in use^2^. The distributions of these elements are even on the entire surface of the briquette. No element is dominant at a position which can inhibit combustion of the fuel briquette at such position.

## Conclusion

The use of bentonite as inorganic binder for briquetting of subbituminous coal and torrefied biomass has been studied. The physicomechanical durability and energy content of the produced briquettes have been evaluated. Bentonite reduced water resistance index of the briquette. The increment in bentonite within the briquette improved the drop to fracture and impact resistance. The highest drop to fracture and impact resistance index for the briquettes were 150 times/2 m and 3000, respectively. The highest cold crushing strength was obtained at 10% bentonite content. Bentonite impaired the energy content of the briquettes. The least energy content (17.68 MJ/kg) was obtained at 10% bentonite. Carbon and other elements were evenly distributed within the briquettes. Based on balance needed between physicomechanical durability and energy content, 2% bentonite is recommended as binder content for briquetting subbituminous coal and torrefied biomass. The briquette produced with 2% coal bentonites are good feedstock for thermal plants and rotary kiln.

## Data Availability

The datasets used and/or analysed during the current study available from the corresponding author on reasonable request.
